# An Unusual Case of Retroperitoneal Plexiform Neurofibromas Found in a Trauma Patient

**DOI:** 10.7759/cureus.12997

**Published:** 2021-01-29

**Authors:** Allen Mao, Hunaid N Rana, Mohamed Elramah, Brett Martin

**Affiliations:** 1 Radiology, USA Health University Hospital, Mobile, USA

**Keywords:** plexiform neurofibroma, neurofibromatosis, nf1, neurofibroma, epidural hematoma

## Abstract

The spectrum of presentation for patients who have neurofibromatosis type 1 (NF1), or von Recklinghausen disease, is very diverse due to a phenomenon known as variable expressivity. Patients may or may not present with cutaneous lesions or central nervous system (CNS) manifestations. However, multiple neurofibromas are the pathognomonic hallmark of NF1. The most common abdominal neoplasm is plexiform neurofibromas that affect the retroperitoneal region. We highlight the hospital course of a patient with an unknown history of NF1 who presented for head trauma with plexiform neurofibromas found incidentally on imaging. The radiographic features of neurofibromas are described in addition to the discussion of management and prognosis of NF1.

## Introduction

Neurofibromatosis type 1 (NF1) is a multisystem neurocutaneous disease that primarily targets the central nervous system (CNS), skin, and eyes. It affects approximately one in every 3,000 individuals and presents with variable expressivity, but almost 100% penetrance by age 5 [1]. Variable expressivity represents the degree in which a genotype is phenotypically expressed, where there is a range of disease severity and symptom presentation. Approximately 50% of patients will have autosomal dominant inheritance, while the other half will have de novo germline mutations in genes involved in the oncogene protein P21/mitogen-activated protein kinase (Ras/MAPK) pathway. There is a strong association of NF1 with neurofibromas or benign peripheral nerve sheath tumors. In particular, plexiform neurofibromas are a variant that targets deeper nerves and can be found in the retroperitoneum. On CT, the masses are characterized by their low density and homogenous appearance. They are particularly difficult to resect and are also known to have the ability to become malignant.

## Case presentation

A 20-year-old Caucasian male with unknown past medical history presents to the emergency department as a trauma transfer from an outside hospital for coup contra-coup injuries sustained as a pedestrian stuck by motor vehicle. He denied loss of consciousness and was able to recall the entire event. The patient states that he was riding his bicycle when he was hit by a car, causing his head to smash against the windshield, and propelling his body over the hood. On arrival, he had a Glasgow Coma Scale (GCS) of 15, and was alert and oriented to person, place, and time. His chief complaint was acute-onset neck pain and headache that was relieved with analgesics.

**Figure 1:**
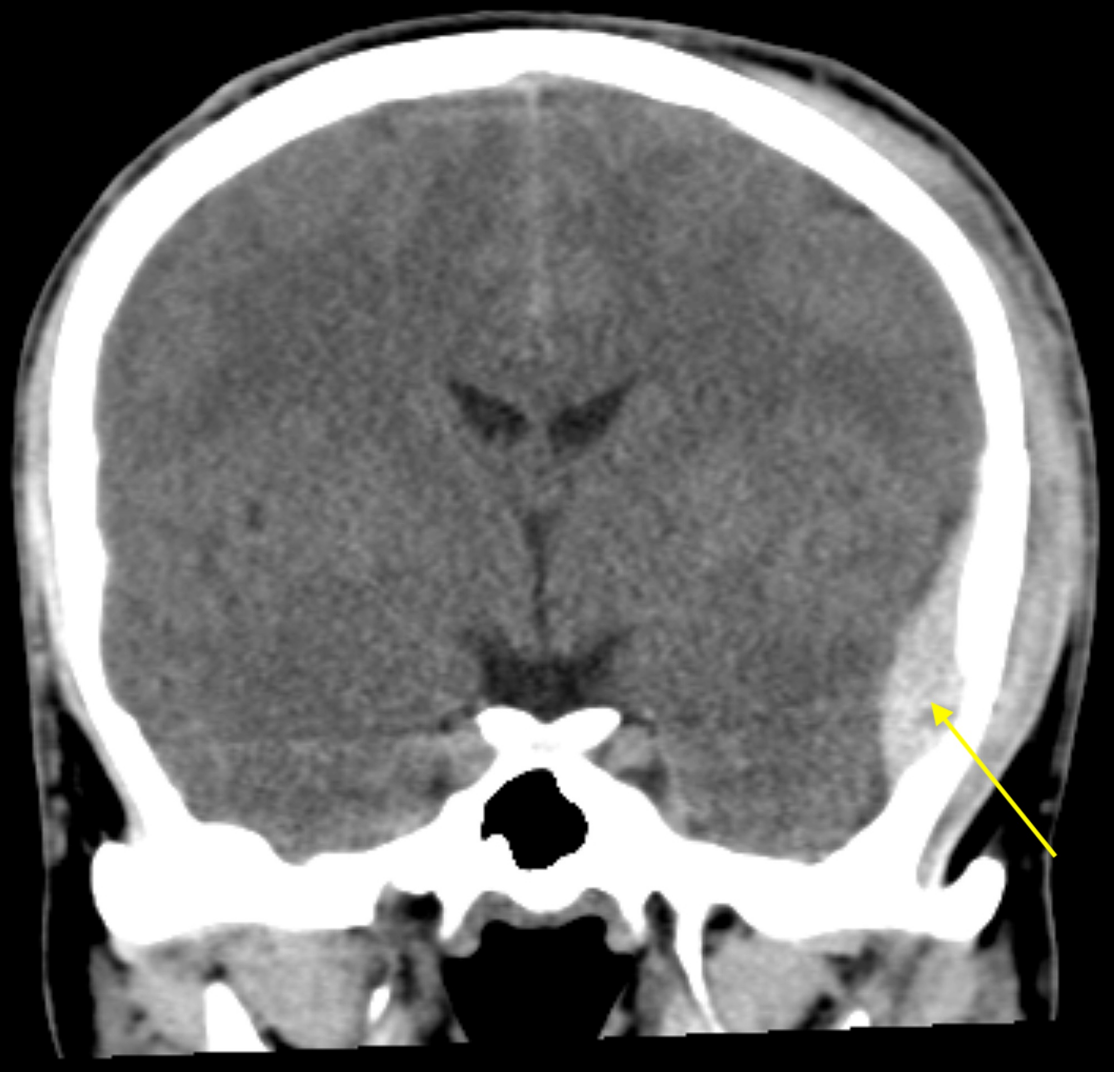
Non-contrasted coronal CT demonstrates hyperdense lentiform epidural hematoma in the left temporal lobe with maximum thickness measuring approximately 1.4 cm (yellow arrow).

**Figure 2:**
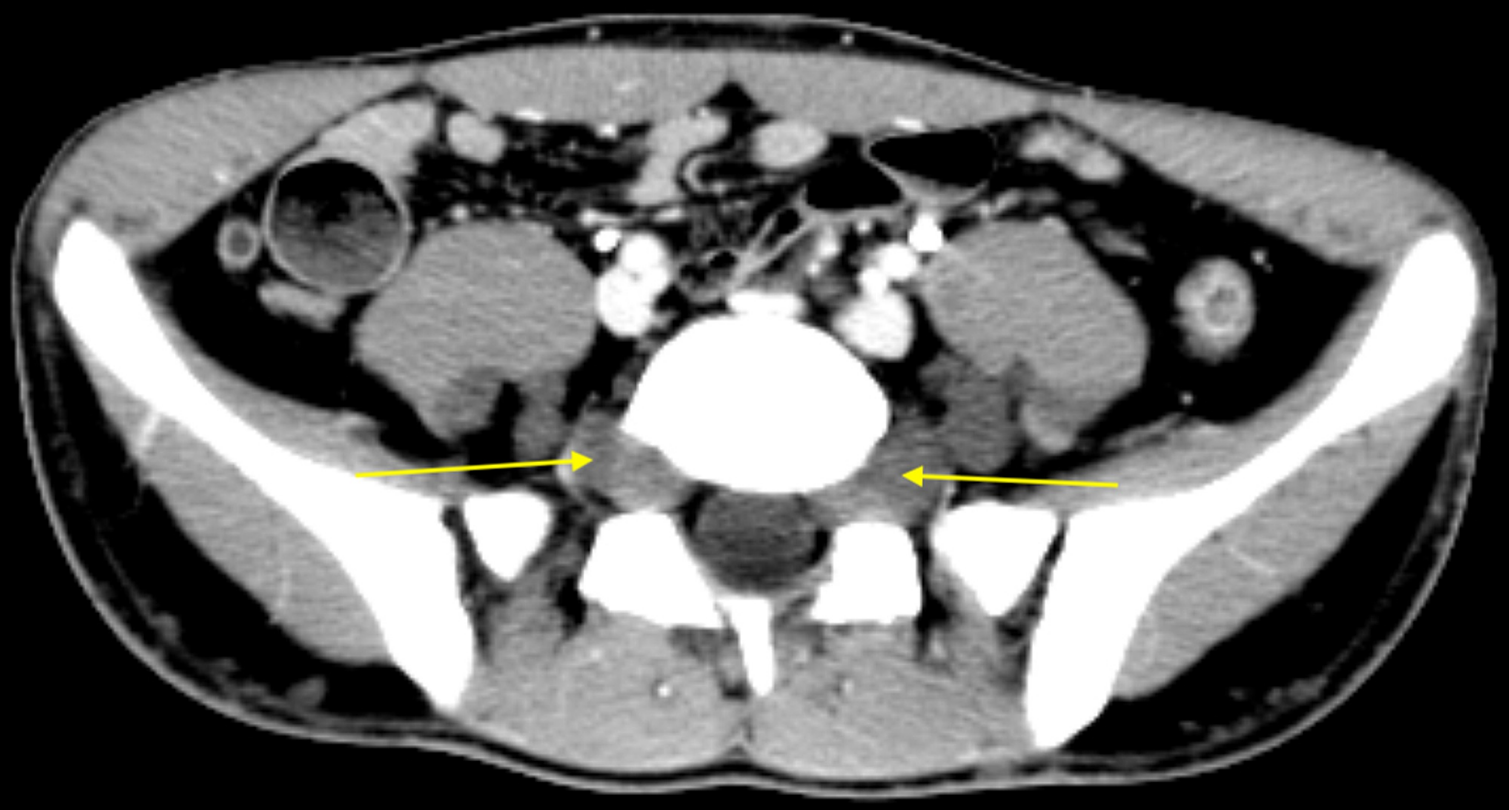
Contrasted axial CT demonstrates bilateral hypoattenuating masses in the parapsoas and presacral regions with homogenous appearance (yellow arrows).

**Figure 3:**
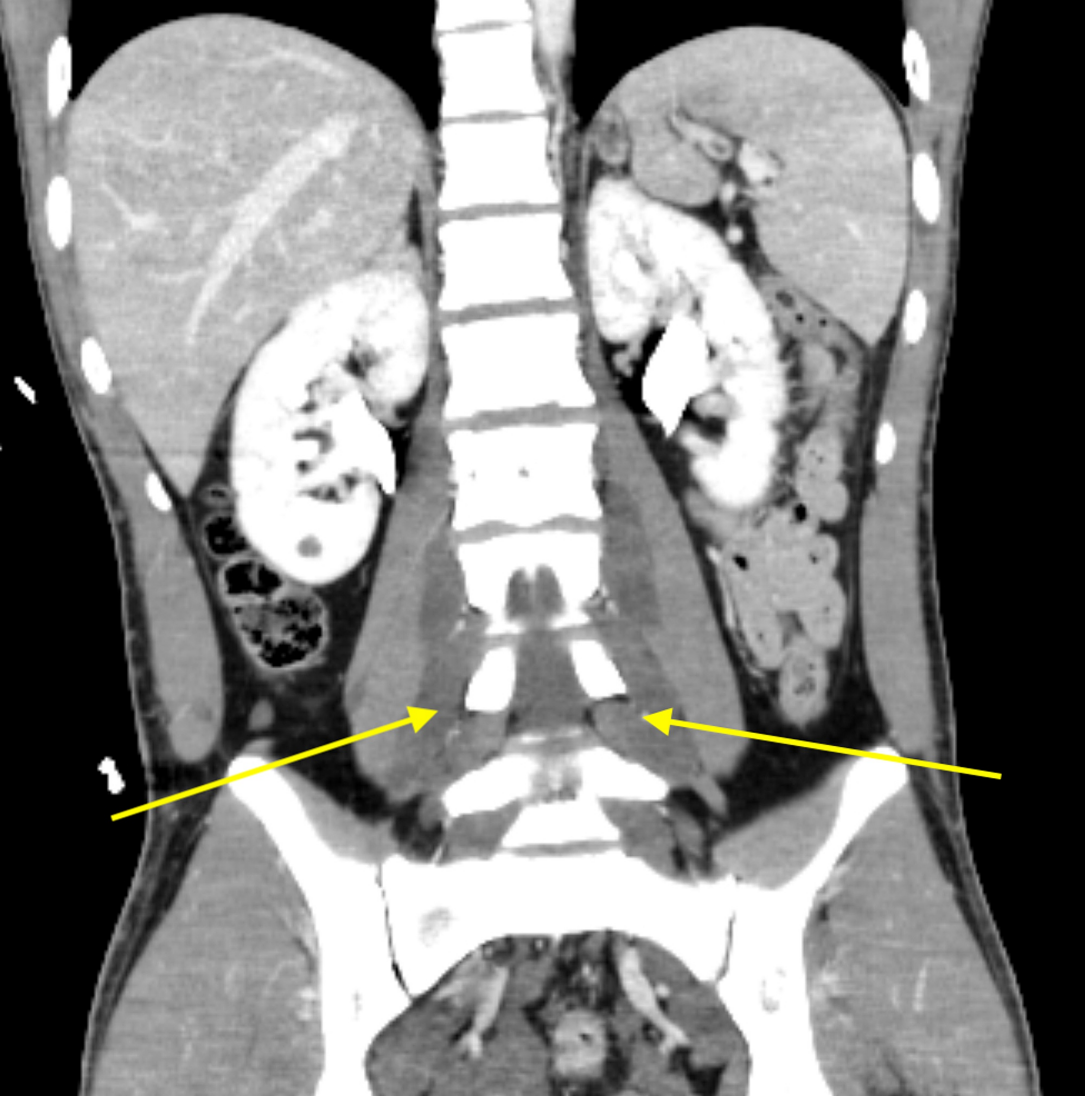
Contrasted coronal CT again demonstrates low-density masses that traverse multiple neuroforamina bilaterally and are characteristic of retroperitoneal plexiform neurofibromas (yellow arrows).

Trauma workup was initiated with whole-body CT for investigation of polytrauma. Initial non-contrasted head CT showed left temporal epidural hematoma measuring 1.4 cm with a lenticular shape (Figure 1). CT abdomen and pelvis with intravenous contrast demonstrated numerous bilateral symmetric hypoattenuating masses exiting the lumbar neuroforamina that tracked along the parapsoas and presacral regions, a finding consistent with retroperitoneal plexiform neurofibromas (Figures 2,3). There were no visible cutaneous or CNS manifestations on physical examination. MRI of the lumbosacral spine with and without contrast was recommended in order to further characterize the lesions but was unable to be safely performed due to the patient’s condition.

Given the CT findings of epidural bleeding, the patient was admitted to the surgical intensive care unit for close monitoring and neurosurgery was consulted. They recommended starting levetiracetam (Keppra) due to increased risk of seizure activity secondary to the location of hemorrhage in the temporal lobe. Serial head CTs were obtained to track daily progression of the intracranial bleeding. The patient was placed in an Aspen cervical collar in order to stabilize the C-spine and to help alleviate neck pain. The following day, head CT displayed worsening of left temporal epidural hematoma.

On day 3 of admission, the patient developed neurological change on exam and was noted to be dysarthric with slurred speech. Due to concern for acute deterioration in the setting of deteriorating epidural hemorrhage on CT, he was taken to the operating room by neurosurgery for emergent craniotomy. Left burr hole craniotomy was performed for evacuation of epidural hemorrhage in the setting of brain compression and dysarthria with the benefit of preventing uncal herniation and potential consequences of elevated intracranial pressures. Post-surgical CT showed expected postoperative pneumocephaly in addition to trace blood products with residual left temporal hygroma. On physical exam, he had improved dysarthria with spontaneous speech and no focal neurological deficits. The patient was transferred from the surgical ICU to the floor and worked with physical therapists for ambulation. Given the patient’s hemodynamic stability, he was discharged on postoperative day 4 with a cervical collar and seven-day course of Keppra for seizure prophylaxis. He was instructed to follow-up with neurosurgery with repeat head CT in addition to further evaluation of NF1 as an outpatient.

## Discussion

NF1 is the most common phakomatosis, or neurocutaneous disorder, that targets organs embryologically derived from ectoderm, including CNS, eyes, and skin. The constellation of symptoms can include neurofibromas, optic gliomas, Lisch nodules, axillary freckling, and café au lait spots. NF1 is classified as a RASopathy, due to the overactivity of the Ras/MAPK pathway from a mutation in the tumor suppression gene neurofibromin on chromosome 17q11.2 [1]. Neurofibromin is a negative regulator of Ras, which leads to unchecked progression of the cell cycle and uncontrolled growth of neurofibroma tumors. Cutaneous neurofibromas can present with pain or cause psychologic impairment due to cosmetic distress.

Plexiform neurofibromas are an uncommon variant of neurofibromas and are characterized by deeper lesions affecting nerves and plexus. Unlike diffuse cutaneous neurofibromas, plexiform variants have an increased risk of malignant transformation and are essentially pathognomonic for NF1, as only one plexiform neurofibroma is sufficient for the diagnosis via National Institute of Health Consensus Criteria [2]. Plexiform neurofibromas can occur in a variety of locations in the body including subcutaneous tissues, mediastinum, paraspinal regions, and retroperitoneum. Patients are usually diagnosed in early childhood and plexiform neurofibromas are found in approximately 30% of patients with NF1 with no gender predilection [3].

Imaging via CT offers a non-invasive method to definitively diagnose retroperitoneal plexiform neurofibromas without putting patients through the risk and pain of an unnecessary biopsy. On CT, lesions appear homogenous, hypoattenuating, and track along the parapsoas and presacral regions in a bilateral and symmetric fashion [4]. The low attenuation has been attributed to a combination of elevated lipid matter from the perineural adipose tissue and increased myxoid stroma. Typically, the masses are not calcified or contained by a discernable capsule. MRI may be able to better identify the neurofibromas by certain signal characteristics. On T1-weighted MRI, the masses appear hypointense, while T2-weight imaging classically displays a rim of hyperintensity surrounding central foci of hypointensity [3]. This target sign on T2 MRI is indicative of a peripheral nerve sheath tumor.

Management of asymptomatic patients consists of serial monitoring of the plexiform neurofibromas for complications like progression to malignancy. In a patient with bilateral retroperitoneal plexiform neurofibromas, the appearance of an asymmetric, unilateral mass can be concerning for a malignant nerve sheath tumor and may prompt further workup with percutaneous biopsy but has potential risks of sensorineural deficits. Overall, the prognosis for NF1 patients can be variable but total life expectancy is typically half of that seen in non-affected individuals. The most common cause of death is attributed to malignant neoplasms or cardiovascular abnormalities such as aneurysms [5].

## Conclusions

We highlight an interesting case of a patient who presented for head trauma who was found to have retroperitoneal plexiform neurofibromas incidentally on whole-body CT. This case emphasizes the wide spectrum that NF1 can present due to variable expressivity. The diagnosis of neurofibromas can be made definitively via CT or MRI imaging, which avoids the unnecessary risk of surgical biopsy. Unless debilitating symptoms are present, neurofibromas are generally treated non-surgically with serial imaging for surveillance.
